# Biochemical interaction of anti-HCV telaprevir with the ABC transporters P-glycoprotein and breast cancer resistance protein

**DOI:** 10.1186/1756-0500-6-445

**Published:** 2013-11-06

**Authors:** Yuria Fujita, Kohji Noguchi, Tomonori Suzuki, Kazuhiro Katayama, Yoshikazu Sugimoto

**Affiliations:** 1 Division of Chemotherapy, Faculty of Pharmacy, Keio University, 1-5-30 Shibakoen, Minato-ku, Tokyo 105-8512, Japan

**Keywords:** P-glycoprotein, Breast cancer resistance protein, Telaprevir, Inhibition, Transporter, Vesicle transporter assay, Kinetics, [^125^I]-IAAP, HCV, Photoaffinity labeling

## Abstract

**Background:**

The ATP-binding cassette (ABC) transporters P-glycoprotein (P-gp)/ABCB1 and breast cancer resistance protein (BCRP)/ABCG2 are involved in the intestinal absorption and renal excretion of various substrate drugs. Their activities affect sub-therapeutic drug concentrations and excretion of natural transporter substrates. The new oral anti-HCV drug telaprevir has dramatically improved the efficacy of hepatitis-C virus (HCV) treatment, and recent studies have suggested a possible pharmacological interaction between telaprevir and P-gp. We studied the kinetics of *in vitro* interactions between telaprevir and P-gp and BCRP to understand the molecular basis of that interaction.

**Findings:**

The effect of telaprevir on P-gp- and BCRP-mediated transport was evaluated by an *in vitro* vesicle transporter assay using different transport substrates, and the kinetics of transporter inhibition was determined. The results showed that telaprevir could inhibit P-gp- and BCRP-mediated transport in the *in vitro* vesicle transport assay, with each IC_50_ values of ≈ 7 μmol/L and ≈ 30 μmol/L, respectively. Analyses of Lineweaver–Burk plots showed that telaprevir was likely to be a competitive inhibitor against P-gp and BCRP. Photoaffinity labeling experiments were employed to observe competitive inhibition by telaprevir using iodoarylazidoprazosin (IAAP) as a binding substrate for P-gp and BCRP. These experiments revealed that telaprevir inhibited [^125^I]-IAAP-binding with P-gp and BCRP.

**Conclusion:**

Telaprevir competitively inhibited P-gp and BCRP, and P-gp-mediated transport was more sensitive to telaprevir compared with BCRP-mediated transport. These data suggest that telaprevir represses the transporter functions of P-gp and BCRP *via* direct inhibition.

## Background

Telaprevir is a new, orally-administered drug acting directly against hepatitis-C virus (HCV) non-structural 3/4A (NS3/4A) protease [1]. Combination of telaprevir, pegylated interferon and ribavirin has been shown to increase significantly the sustained virological response in patients [2]. Antiviral chemotherapies for the HCV, hepatitis-B virus (HBV) and human immunodeficiency virus (HIV) comprise various drug combinations, so pharmacological drug–drug interactions with members of the cytochrome P-450 superfamily have been considered carefully [3]. With regard to drug interactions with transporters, functional interactions between anti-HCV drugs and the ABC transporter protein have become apparent [4,5]. Telaprevir is reported to be a substrate and an inhibitor of P-gp, but not of breast cancer resistance protein (BCRP) [6] (available at http://pi.vrtx.com/files/uspi_telaprevir.pdf; 2011); however, the biochemical basis of such differences in inhibition is incompletely understood. Moreover, understanding based on observations from clinical and cell-based assays should include direct and indirect effects by metabolic enzymes [7-9]. Therefore, studies on the direct effects between drugs and transporters are necessary to understand the mechanism of complicated drug–drug interactions.

We have been studying various drug interactions with P-gp and BCRP [10-16]. For example, verapamil and tyrosine kinase inhibitors (TKIs) such as imatinib, gefitinib, and erlotinib could be inhibitors of P-gp. Fumitremorgin C (FTC), certain flavonoids and the TKIs mentioned above also inhibit BCRP-mediated transport. Our studies have suggested that multiple binding sites on ABC transporter proteins cause complicated differences in transporter inhibition, depending on the substrate and inhibitor [15,17,18].

In the present study, we investigated the biochemical basis of the *in vitro* interactions of P-gp and BCRP with telaprevir. We analyzed their kinetics using *in vitro* cell-free systems to provide mechanistic insights into the interaction between ABC transporters and telaprevir.

## Methods

### Reagents

Telaprevir was purchased from Selleck Chemicals (Houston, TX, USA). FTC was from Alexis (San Diego, CA, USA) and verapamil was from Sigma–Aldrich (St. Louis, MO, USA). [^3^H]-labeled vincristine (VCR), estrone 3-sulfate (E1S), methotrexate (MTX), and [^125^I]-labeled iodoarylazidoprazosin (IAAP) were purchased from Perkin-Elmer Life Sciences (Boston, MA, USA). All other reagents were available commercially.

### Intravesicular transport assay

Plasma membrane vesicles (22.5 μg of protein) were prepared from K562/MDR and K562/BCRP cells as described previously [13]. Then, they were mixed with [^3^H]-labeled VCR (for P-gp) or E1S or MTX (for BCRP), and telaprevir at the indicated concentration in the reaction buffer (0.25 mol/L sucrose, 10 mmol/L HEPES-NaOH (pH 7.4), 10 mmol/L MgCl_2_, 10 mmol/L phosphocreatine, 100 μg/mL creatine phosphokinase, with or without 3 mmol/L adenosine triphosphate (ATP)) in a total volume of 50 μL. After 10 min of incubation at 25°C, the reaction mixture was stopped by the addition of ice-cold stop solution (0.25 mol/L sucrose, 10 mmol/L HEPES-NaOH (pH 7.4), 0.1 mol/L NaCl) and centrifuged at 18,000 × *g* for 10 min at 4°C. The pellets were solubilized by a liquid scintillation counter to measure their radioactivity levels. For kinetics analyses, the Michaelis–Menten equation and Lineweaver–Burk plots were applied to visualize the mechanism of transport inhibition, as v = *Vmax*[S]/([S] + (1 + [I]/*Ki*)Km), where [S] and [I] are concentrations of substrate and inhibitor respectively, and v is velocity of substrate uptake by transporter. The enzymatic values for Michaelis–Menten constants of substrates (*Km*) and inhibitors (*Ki*) and the maximum uptake rate for a transporter-mediated process (*Vmax*) were calculated by estimating the slopes (=*Km*(1 + [I]/*Ki*)/*Vmax*), *x*-intercepts (= - 1/(*Km*(1 + [I]/*Ki*)) and *y*-intercepts (=1/*Vmax*) on the plots mentioned above in competitive-inhibition mode. Competitive inhibition causes different slopes and *x*-intercepts between the two data, but with similar *y*-intercepts. Non-competitive inhibition shows a similar *x*-intercept but different slopes and *y*-intercepts. Uncompetitive inhibition shows similar slopes.

### Photoaffinity labeling with IAAP

Photoaffinity labeling assay was done as described previously [13]. In brief, plasma membrane fractions (45 μg protein/sample) were pre-incubated at 4°C for 5 min at the indicated concentration of telaprevir in 0.25 mol/L sucrose solution containing 10 mmol/L HEPES-NaOH (pH 7.4). Then, 5 nmol/L radioactive iodine-labeled IAAP, i.e., [^125^I]-IAAP (2200 Ci/mmol), was added and the mixture incubated for an additional 10 min. Samples were kept on ice and illuminated with an ultraviolet lamp (B-100AP; UVP, Upland, CA, USA) at 365 nm for 30 min. These processes were carried out on ice. For the BCRP assay, the labeled BCRP protein was solubilized in a buffer (1% NP-40, 20 mmol/L Tris–HCl (pH 7.4), 150 mmol/L NaCl, 1 mmol/L ethylenediamine tetra-acetic acid (EDTA)) and immunoprecipitated with anti-BCRP antibody BXP-21 (Millipore, Billerica, MA, USA), then solubilized by Laemmli sodium dodecyl sulfate (SDS) buffer. For the P-gp assay, membrane fractions were solubilized directly by Laemmli SDS buffer. Samples were separated by sodium dodecyl sulfate-polyacrylamide gel electrophoresis (SDS-PAGE), and gels were dried. The binding of [^125^I]-IAAP with P-gp and BCRP was quantified using a FLA7000 Bioimage Analyzer (Fuji Film, Tokyo, Japan) and Multi-Gauge software (Fuji Film).

## Findings

### Inhibition of P-gp and BCRP by telaprevir in vitro

The intravesicular transport assay was carried out to analyze the kinetics of telaprevir inhibition on P-gp- and BCRP-mediated transport *in vitro*.

**Figure 1:**
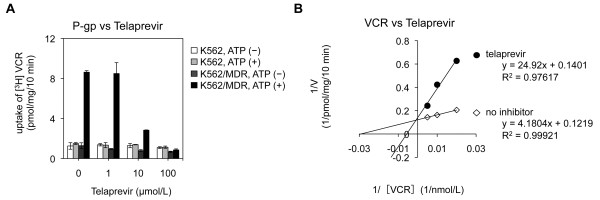
**Effect of telaprevir on the intravesicular transport of vincristine sulfate (VCR) by P-gp. A**. The ability to transport VCR in telaprevir-absent or -present conditions was determined by measuring the radioactivity taken up in membrane vesicles. **B**. Analyses of Lineweaver–Burk plots of inhibition of VCR uptake by P-gp. The VCR concentration was 100 nmol/L **(A)** and 50, 100, and 200 nmol/L **(B)**, and of telaprevir was 0, 1, 10, and 100 μmol/L **(A)** and 20 μmol/L **(B)**, respectively. Membrane vesicles from K562/MDR cells were mixed with each concentration of VCR, telaprevir, and 3 mmol/L of ATP in the incubation medium as described in the Methods section. VCR uptake is shown for parental K562 (white column for ATP-absent; gray column for ATP-present) and K562⁄MDR (dark-gray column for ATP-absent; black column for ATP-present) **(A)** and the inverse is shown for no inhibitor (open rhombus) and with telaprevir (black circle) **(B)**. Results are means ± SD of triplicate **(A)** or quadruplicate **(B**) determinations.

Figure 1A shows that telaprevir inhibited ATP-dependent [^3^H]-VCR transport *in vitro* in a dose-dependent manner. Analyses of Lineweaver–Burk plots showed that the inhibitory mode of telaprevir for P-gp-mediated VCR transport was competitive (Figure 1B). The calculated *Vmax* values (pmol/mg/10 min) were 8.2 in the control condition and 7.1 in the presence of telaprevir (20 μmol/L). The calculated *Ki* value of telaprevir on P-gp-mediated VCR transport was 4.8 μmol/L (Table 1). These results suggested that telaprevir acted as a competitive inhibitor for P-gp-mediated VCR transport.

**Table 1:** Analyses of Lineweaver–Burk plots for P-gp

***Vmax *** **(pmol/mg/10 min)**		***Ki *** **(μmol/L)**
**No inhibitor**	**With 20 μmol/L telaprevir**	
8.2	7.1	4.8

**Figure 2:**
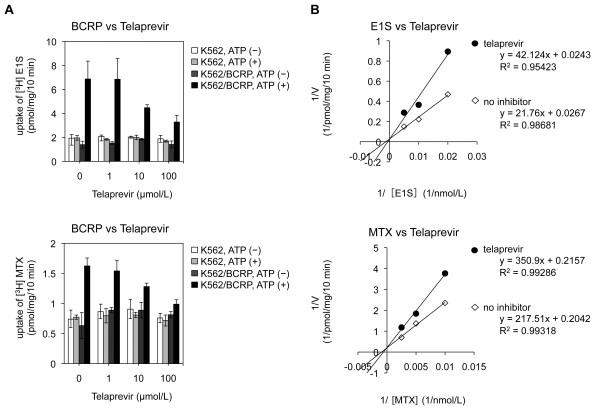
**Effect of telaprevir on the intravesicular transport of estrone 3-sulfate (E1S) and MTX by BCRP. A**. The ability to transport E1S at telaprevir-absent or -present conditions was determined by measuring the radioactivity of [^3^H]-E1S taken up in membrane vesicles. A similar experiment was done for MTX. **B**. Analyses of Lineweaver–Burk plots of inhibition of E1S and MTX uptake by BCRP. E1S concentration was 50 nmol/L **(A)** and 50, 100, and 200 nmol/L **(B)**, and of telaprevir was 0, 1, 10, and 100 μmol/L **(A)** and 50 μmol/L **(B)**, respectively. MTX concentration was 100 nmol/L **(A)**, and 100, 200 and 400 nmol/L (B). Membrane vesicles of K562/BCRP cells were used. The procedures were almost identical to those shown in Figure 1.

Inhibitory effect of telaprevir on BCRP-mediated transport *in vitro* was also analyzed using two transport substrates: E1S and MTX. Telaprevir inhibited [^3^H]-E1S and [^3^H]-MTX transport in a dose-dependent manner at comparable levels (Figure 2A). Analyses of Lineweaver–Burk plots showed that the inhibitory mode of telaprevir for BCRP-mediated E1S and MTX transports appeared to be competitive (Figure 2B). The calculated *Vmax* values (pmol/mg/10 min) were 38 in the control condition and 41 in the presence of telaprevir (50 μmol/L). Similar analysis on BCRP-mediated MTX transport also demonstrated that the calculated *Vmax* values (pmol/mg/10 min) were 4.9 in the control condition and 4.6 in the presence of telaprevir (50 μmol/L). The calculated *Ki* value of telaprevir on BCRP-mediated E1S transport was 44 μmol/L, and that on BCRP-mediated MTX transport was 94.8 μmol/L; these findings suggested that BCRP was more resistant to telaprevir than P-gp (Table 2).

**Table 2:** Analyses of Lineweaver–Burk plots for BCRP

	***Vmax *** **(pmol/mg/10 min)**		***Ki *** **(μmol/L)**
	**No inhibitor**	**With 50 μmol/L telaprevir**	
E1S	37.5	41.2	44.4
MTX	4.9	4.6	94.8

The calculated half-maximal inhibitory concentration (IC_50_) values of telaprevir for each substrate transport are summarized in Table 3. The values suggested that a higher concentration (≈30 μmol/L) of telaprevir was required to elicit 50% inhibition of BCRP-mediated transport compared with that of P-gp-mediated transport (7 μmol/L). These results suggested that the inhibitory kinetics of telaprevir on the transporter function of BCRP and P-gp were likely to be competitive.

**Table 3:** **IC**_**50 **_**values of telaprevir on transport activity by P-gp or BCRP**

	**Substrate**	**IC**_**50 **_**of telaprevir (μmol/L)**
P-gp	VCR	7
BCRP	E1S	30
	MTX	31

### Effects of telaprevir on IAAP-binding to P-gp and BCRP

**Figure 3:**
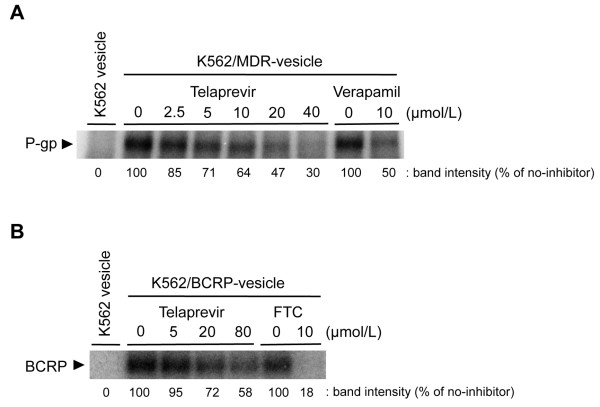
**Effect of telaprevir on photoaffinity labeling of P-gp and BCRP with [**^**125**^**I]-IAAP.** K562/MDR- or K562/BCRP-vesicles were mixed with the indicated concentrations of telaprevir for 5 min. Vesicles were pre-incubated with 5 nmol/L [^125^I]-IAAP (2200 Ci/mmol) for 10 min, and then illuminated with a UV lamp (365 nm) for 30 min. IAAP-labeled protein was solubilized by Laemmli SDS buffer directly (for the P-gp assay) or after immunoprecipitation (for the BCRP assay), and subjected to SDS-PAGE. Signals were visualized and analyzed with a FLA7000 system. The black arrowheads are P-gp or BCRP. Verapamil and FTC (typical inhibitors of P-gp or BCRP, respectively) were used as controls. The binding of [^125^I]-IAAP with P-gp and BCRP was calculated from standard curve analysis using a FLA7000 Bioimage Analyzer (Fuji Film, Tokyo, Japan) and Multi-Gauge software (Fuji Film).

The data mentioned above could not be used to ascertain if telaprevir competed with substrate binding on the transporters. Thus, we examined the effect of telaprevir on [^125^I]-IAAP-binding with P-gp and BCRP by a photoaffinity labeling experiment to observe the direct competition of telaprevir with the transporter substrate IAAP. Telaprevir apparently suppressed [^125^I]-IAAP-binding with P-gp in a dose-dependent manner (Figures 3A and B). In contrast, [^125^I]-IAAP-binding with BCRP was relatively resistant to telaprevir than that with P-gp, and a higher concentration of telaprevir was required for the inhibition of IAAP-binding with BCRP. The calculated IC_50_ values were ≈ 17 μmol/L for P-gp and >80 μmol/L for BCRP (Table 4). Overall, our results revealed that telaprevir would be a direct competitive inhibitor for P-gp and BCRP, and that P-gp would be a better target for telaprevir than BCRP.

**Table 4:** **IC**_**50 **_**values of telaprevir on [**^**125**^**I]-IAAP binding with P-gp or BCRP**

	**IC**_**50 **_**of telaprevir (μmol/L)**
P-gp	16.5
BCRP	>80

## Discussion

Telaprevir has been reported to be a substrate and inhibitor of P-gp [6]. Recent clinical pharmacokinetic and pharmacodynamic studies suggested enhanced absorption of digoxin by inhibition or saturation of P-gp in the intestine due to telaprevir in the gut [7,8]. Such studies suggested that telaprevir is a substrate and inhibitor of CYP 3A4 and P-gp *in vivo*. However, biochemical analyses are needed to clarify the mechanism of biochemical interaction between telaprevir and P-gp.

Our previous studies on ABC transporters revealed that verapamil and TKIs (including imatinib, gefitinib, erlotinib and sunitinib) are inhibitors of P-gp as competitive inhibitors [13-15]. In addition, we showed that FTC, certain flavonoids and the TKIs mentioned above are potential competitive inhibitors of BCRP [12]. In the present study, we demonstrated that telaprevir is a potential direct inhibitor of P-gp and BCRP. Moreover, to provide mechanistic insights for understanding the interaction between ABC transporters and telaprevir, our analyses of photoaffinity labeling and kinetics showed, for the first time, that telaprevir could be a direct competitive inhibitor for P-gp and BCRP. *In vitro* cell-free experiments showed that the IC_50_ value of telaprevir-mediated P-gp inhibition was as low as 7 μmol/L. This concentration would be reached in the gut after oral administration of telaprevir. In contrast, BCRP-mediated transport was relatively insensitive to telaprevir, and a higher concentration of telaprevir was required for inhibition of IAAP-binding with BCRP. The HCV protease inhibitor boceprevir has also been reported to interact with P-gp and BCRP using a cellular system [9,19]. However, the inhibitory effect of boceprevir on P-gp seemed to be very low, and that on BCRP was shown to be weak (IC_50_ ≈ 80 μM) in that study. Combined with the data in the present study, telaprevir appears to be a strong inhibitor of P-gp, and we hypothesize that intestinal P-gp would be a pharmacological target for telaprevir.

Similar inhibitory potential of telaprevir on other transporters has been characterized (albeit examined by experimental cellular systems). This characterization has been mainly on the renal transporters OCT2 and MATE1 with IC_50_ values (μmol/L) of 6.4 and 23.0, and hepatic transporters OATP1B1, OATP1B and OCT1, with IC_50_ values of 2.2, 36.8 and 20.7, respectively [8]. However, the renal clearance of digoxin (a P-gp substrate) has been reported to be similar to that without telaprevir [7]. Hence, the effect of telaprevir on P-gp activity in the kidney seems to be minimal. We suspect that the steady-state concentration of telaprevir in each tissue may be an important factor modulating the activities of P-gp and other transporters.

Our data revealed that telaprevir-mediated P-gp inhibition would be mediated by a direct competitive mechanism. Hence, the combination of substrate and inhibitor could make prediction of drug–drug interactions on P-gp in each setting more difficult. Indeed, many patients suffering from HCV and HIV might be subjected to anti-HIV drugs and anti-HCV drugs, and many of those antiviral drugs are known to interact with CYPs and transporter proteins. Multiple binding sites are suggested on ABC transporter proteins [18], so a complicated drug combination could cause complex drug–drug interactions by complicated transporter inhibition mechanism. Thus, detailed analyses for each drug based on cell-free systems could contribute to better understanding of the mechanisms of drug–transporter interactions, particularly those in combination chemotherapy.

## Conclusions

Telaprevir can inhibit P-gp- and BCRP-mediated transport, and P-gp-mediated transport is more sensitive to telaprevir-mediated inhibition. Our study will be helpful for theoretical consideration of interactions between intestinal P-gp and telaprevir during long-term anti-HCV chemotherapy.

## Abbreviations

ABC: ATP-binding cassette; BCRP: Breast cancer resistance protein; IC50: 50% inhibitory concentration.

## Competing interests

The authors declare that they have no competing interests.

## Authors’ contributions

YF, KN, TS and KK carried out experiments and data analyses. KN and YS designed the study and supervised the data. YF, KN and YS wrote the manuscript. All authors approved the final manuscript.
